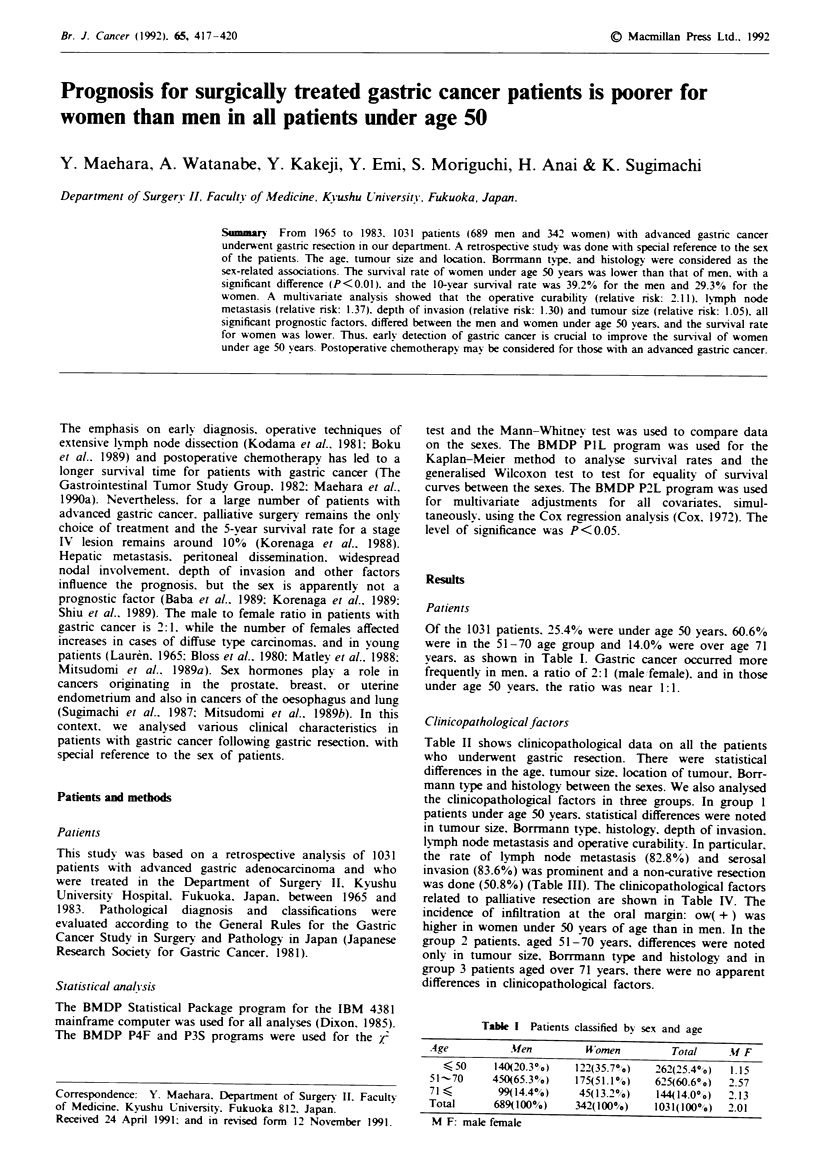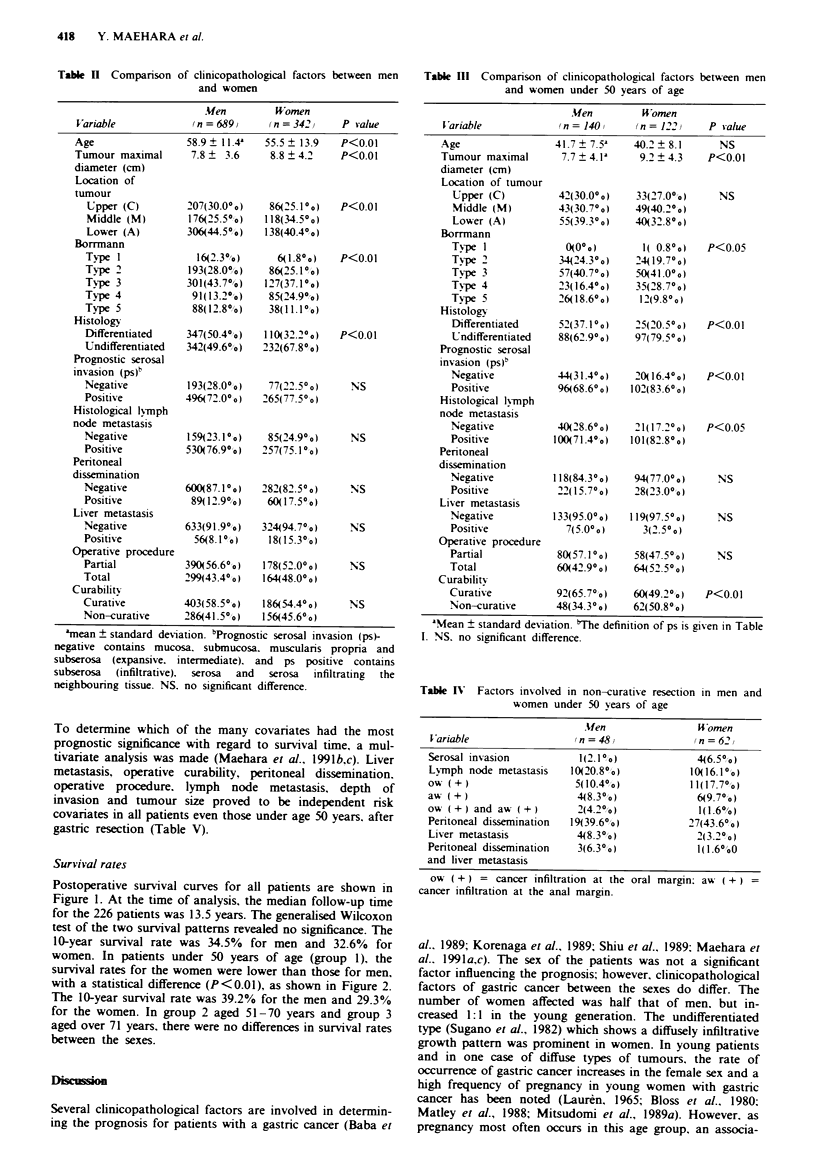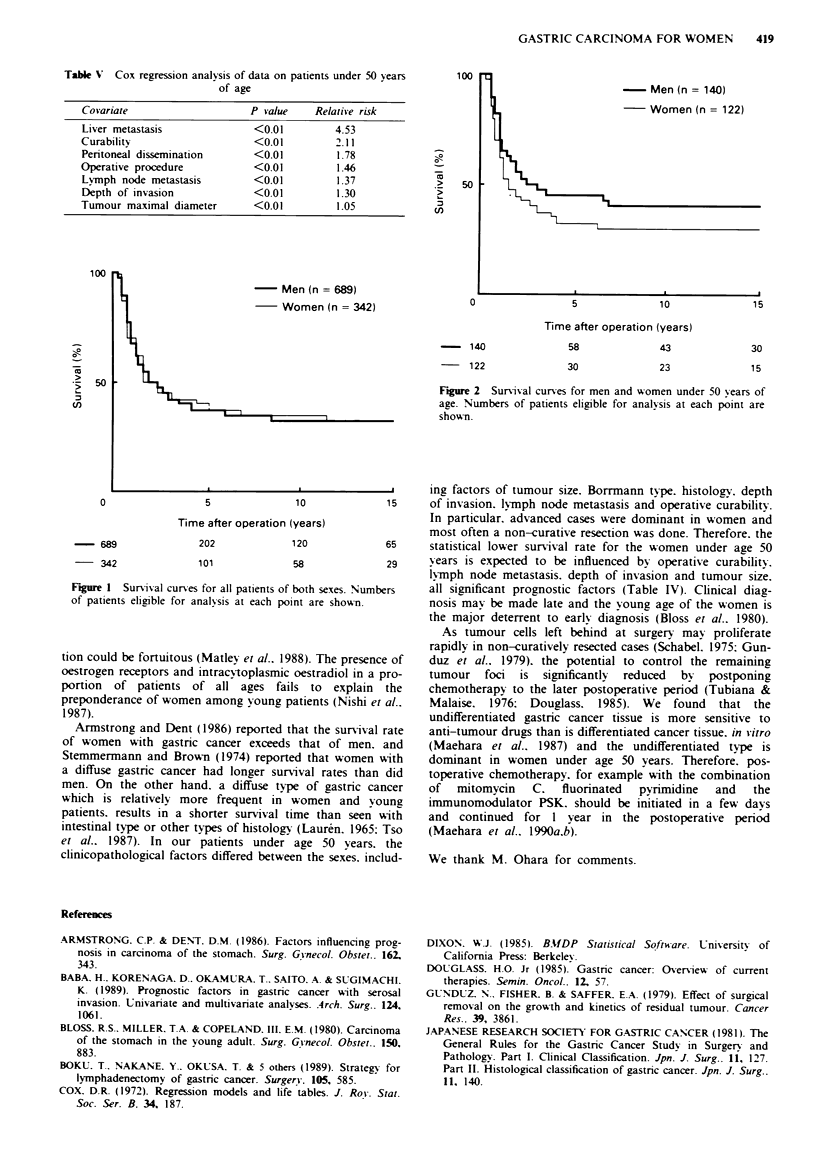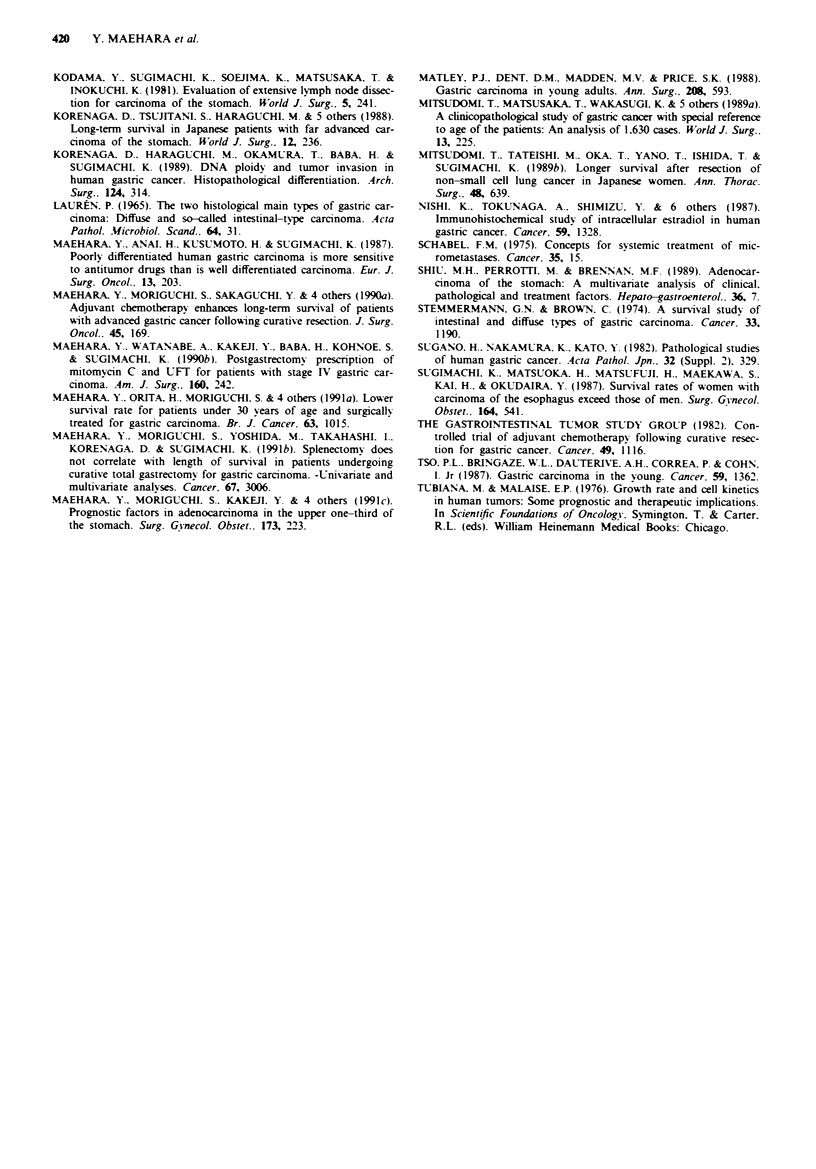# Prognosis for surgically treated gastric cancer patients is poorer for women than men in all patients under age 50.

**DOI:** 10.1038/bjc.1992.85

**Published:** 1992-03

**Authors:** Y. Maehara, A. Watanabe, Y. Kakeji, Y. Emi, S. Moriguchi, H. Anai, K. Sugimachi

**Affiliations:** Department of Surgery II, Faculty of Medicine, Kyushu University, Fukuoka, Japan.

## Abstract

From 1965 to 1983, 1031 patients (689 men and 342 women) with advanced gastric cancer underwent gastric resection in our department. A retrospective study was done with special reference to the sex of the patients. The age, tumour size and location, Borrmann type, and histology were considered as the sex-related associations. The survival rate of women under age 50 years was lower than that of men, with a significant difference (P less than 0.01), and the 10-year survival rate was 39.2% for the men and 29.3% for the women. A multivariate analysis showed that the operative curability (relative risk: 2.11), lymph node metastasis (relative risk: 1.37), depth of invasion (relative risk: 1.30) and tumour size (relative risk: 1.05), all significant prognostic factors, differed between the men and women under age 50 years, and the survival rate for women was lower. Thus, early detection of gastric cancer is crucial to improve the survival of women under age 50 years. Postoperative chemotherapy may be considered for those with an advanced gastric cancer.


Br. J. Cancer (1992), 65, 417-420 © Macmillan Press Ltd., 1992

### Prognosis for surgically treated gastric cancer patients is poorer for women than men in all patients under age 50

Y. Maehara, A. Watanabe, Y. Kakeji, Y. Emi, S. Moriguchi, H. Anai & K. Sugimachi

*Department of Surgery II, Faculty of Medicine, Kyushu University, Fukuoka, Japan.*

**Summary** From 1965 to 1983, 1031 patients (689 men and 342 women) with advanced gastric cancer underwent gastric resection in our department. A retrospective study was done with special reference to the sex of the patients. The age, tumour size and location, Borrmann type, and histology were considered as the sex-related associations. The survival rate of women under age 50 years was lower than that of men, with a significant difference ($P<0.01$), and the 10-year survival rate was 39.2% for the men and 29.3% for the women. A multivariate analysis showed that the operative curability (relative risk: 2.11), lymph node metastasis (relative risk: 1.37), depth of invasion (relative risk: 1.30) and tumour size (relative risk: 1.05), all significant prognostic factors, differed between the men and women under age 50 years, and the survival rate for women was lower. Thus, early detection of gastric cancer is crucial to improve the survival of women under age 50 years. Postoperative chemotherapy may be considered for those with an advanced gastric cancer.

The emphasis on early diagnosis, operative techniques of extensive lymph node dissection (Kodama *et al.*, 1981; Boku *et al.*, 1989) and postoperative chemotherapy has led to a longer survival time for patients with gastric cancer (The Gastrointestinal Tumor Study Group, 1982; Maehara *et al.*, 1990a). Nevertheless, for a large number of patients with advanced gastric cancer, palliative surgery remains the only choice of treatment and the 5-year survival rate for a stage IV lesion remains around 10% (Korenaga *et al.*, 1988). Hepatic metastasis, peritoneal dissemination, widespread nodal involvement, depth of invasion and other factors influence the prognosis, but the sex is apparently not a prognostic factor (Baba *et al.*, 1989; Korenaga *et al.*, 1989; Shiu *et al.*, 1989). The male to female ratio in patients with gastric cancer is 2:1, while the number of females affected increases in cases of diffuse type carcinomas, and in young patients (Lauren, 1965; Bloss *et al.*, 1980; Matley *et al.*, 1988; Mitsudomi *et al.*, 1989*a*). Sex hormones play a role in cancers originating in the prostate, breast, or uterine endometrium and also in cancers of the oesophagus and lung (Sugimachi *et al.*, 1987; Mitsudomi *et al.*, 1989*b*). In this context, we analysed various clinical characteristics in patients with gastric cancer following gastric resection, with special reference to the sex of patients.

### Patients and methods

### Patients

This study was based on a retrospective analysis of 1031 patients with advanced gastric adenocarcinoma and who were treated in the Department of Surgery II, Kyushu University Hospital, Fukuoka, Japan, between 1965 and 1983. Pathological diagnosis and classifications were evaluated according to the General Rules for the Gastric Cancer Study in Surgery and Pathology in Japan (Japanese Research Society for Gastric Cancer, 1981).

### Statistical analysis

The BMDP Statistical Package program for the IBM 4381 mainframe computer was used for all analyses (Dixon, 1985). The BMDP P4F and P3S programs were used for the $\chi^2$ test and the Mann–Whitney test was used to compare data on the sexes. The BMDP P1L program was used for the Kaplan–Meier method to analyse survival rates and the generalised Wilcoxon test to test for equality of survival curves between the sexes. The BMDP P2L program was used for multivariate adjustments for all covariates, simultaneously, using the Cox regression analysis (Cox, 1972). The level of significance was $P<0.05$.

### Results

### Patients

Of the 1031 patients, 25.4% were under age 50 years, 60.6% were in the 51–70 age group and 14.0% were over age 71 years, as shown in Table I. Gastric cancer occurred more frequently in men, a ratio of 2:1 (male/female), and in those under age 50 years, the ratio was near 1:1.

### Clinicopathological factors

Table II shows clinicopathological data on all the patients who underwent gastric resection. There were statistical differences in the age, tumour size, location of tumour, Borrmann type and histology between the sexes. We also analysed the clinicopathological factors in three groups. In group 1 patients under age 50 years, statistical differences were noted in tumour size, Borrmann type, histology, depth of invasion, lymph node metastasis and operative curability. In particular, the rate of lymph node metastasis (82.8%) and serosal invasion (83.6%) was prominent and a non-curative resection was done (50.8%) (Table III). The clinicopathological factors related to palliative resection are shown in Table IV. The incidence of infiltration at the oral margin: ow(+) was higher in women under 50 years of age than in men. In the group 2 patients, aged 51–70 years, differences were noted only in tumour size, Borrmann type and histology and in group 3 patients aged over 71 years, there were no apparent differences in clinicopathological factors.

**Table I** Patients classified by sex and age

| *Age* | *Men* | *Women* | *Total* | *M/F* |
|---|---|---|---|---|
| ≤50 | 140(20.3%) | 122(35.7%) | 262(25.4%) | 1.15 |
| 51~70 | 450(65.3%) | 175(51.1%) | 625(60.6%) | 2.57 |
| 71≤ | 99(14.4%) | 45(13.2%) | 144(14.0%) | 2.13 |
| Total | 689(100%) | 342(100%) | 1031(100%) | 2.01 |

M/F: male/female

Correspondence: Y. Maehara, Department of Surgery II, Faculty of Medicine, Kyushu University, Fukuoka 812, Japan.
Received 24 April 1991; and in revised form 12 November 1991.

**Table II** Comparison of clinicopathological factors between men and women

| Variable | Men (n = 689) | Women (n = 342) | P value |
|---|---|---|---|
| Age | 58.9 ± 11.4[a] | 55.5 ± 13.9 | P<0.01 |
| Tumour maximal diameter (cm) | 7.8 ± 3.6 | 8.8 ± 4.2 | P<0.01 |
| Location of tumour | | | |
| Upper (C) | 207(30.0%) | 86(25.1%) | P<0.01 |
| Middle (M) | 176(25.5%) | 118(34.5%) | |
| Lower (A) | 306(44.5%) | 138(40.4%) | |
| Borrmann | | | |
| Type 1 | 16(2.3%) | 6(1.8%) | P<0.01 |
| Type 2 | 193(28.0%) | 86(25.1%) | |
| Type 3 | 301(43.7%) | 127(37.1%) | |
| Type 4 | 91(13.2%) | 85(24.9%) | |
| Type 5 | 88(12.8%) | 38(11.1%) | |
| Histology | | | |
| Differentiated | 347(50.4%) | 110(32.2%) | P<0.01 |
| Undifferentiated | 342(49.6%) | 232(67.8%) | |
| Prognostic serosal invasion (ps)[b] | | | |
| Negative | 193(28.0%) | 77(22.5%) | NS |
| Positive | 496(72.0%) | 265(77.5%) | |
| Histological lymph node metastasis | | | |
| Negative | 159(23.1%) | 85(24.9%) | NS |
| Positive | 530(76.9%) | 257(75.1%) | |
| Peritoneal dissemination | | | |
| Negative | 600(87.1%) | 282(82.5%) | NS |
| Positive | 89(12.9%) | 60(17.5%) | |
| Liver metastasis | | | |
| Negative | 633(91.9%) | 324(94.7%) | NS |
| Positive | 56(8.1%) | 18(15.3%) | |
| Operative procedure | | | |
| Partial | 390(56.6%) | 178(52.0%) | NS |
| Total | 299(43.4%) | 164(48.0%) | |
| Curability | | | |
| Curative | 403(58.5%) | 186(54.4%) | NS |
| Non-curative | 286(41.5%) | 156(45.6%) | |

[a]mean ± standard deviation. [b]Prognostic serosal invasion (ps)-negative contains mucosa, submucosa, muscularis propria and subserosa (expansive, intermediate), and ps positive contains subserosa (infiltrative), serosa and serosa infiltrating the neighbouring tissue. NS, no significant difference.

To determine which of the many covariates had the most prognostic significance with regard to survival time, a multivariate analysis was made (Maehara *et al.*, 1991*b,c*). Liver metastasis, operative curability, peritoneal dissemination, operative procedure, lymph node metastasis, depth of invasion and tumour size proved to be independent risk covariates in all patients even those under age 50 years, after gastric resection (Table V).

*Survival rates*

Postoperative survival curves for all patients are shown in Figure 1. At the time of analysis, the median follow-up time for the 226 patients was 13.5 years. The generalised Wilcoxon test of the two survival patterns revealed no significance. The 10-year survival rate was 34.5% for men and 32.6% for women. In patients under 50 years of age (group 1), the survival rates for the women were lower than those for men, with a statistical difference ($P<0.01$), as shown in Figure 2. The 10-year survival rate was 39.2% for the men and 29.3% for the women. In group 2 aged 51–70 years and group 3 aged over 71 years, there were no differences in survival rates between the sexes.

### Discussion

Several clinicopathological factors are involved in determining the prognosis for patients with a gastric cancer (Baba *et al.*, 1989; Korenaga *et al.*, 1989; Shiu *et al.*, 1989; Maehara *et al.*, 1991*a,c*). The sex of the patients was not a significant factor influencing the prognosis; however, clinicopathological factors of gastric cancer between the sexes do differ. The number of women affected was half that of men, but increased 1:1 in the young generation. The undifferentiated type (Sugano *et al.*, 1982) which shows a diffusely infiltrative growth pattern was prominent in women. In young patients and in one case of diffuse types of tumours, the rate of occurrence of gastric cancer increases in the female sex and a high frequency of pregnancy in young women with gastric cancer has been noted (Laurèn, 1965; Bloss *et al.*, 1980; Matley *et al.*, 1988; Mitsudomi *et al.*, 1989*a*). However, as pregnancy most often occurs in this age group, an associa-

**Table III** Comparison of clinicopathological factors between men and women under 50 years of age

| Variable | Men (n = 140) | Women (n = 122) | P value |
|---|---|---|---|
| Age | 41.7 ± 7.5[a] | 40.2 ± 8.1 | NS |
| Tumour maximal diameter (cm) | 7.7 ± 4.1[a] | 9.2 ± 4.3 | P<0.01 |
| Location of tumour | | | |
| Upper (C) | 42(30.0%) | 33(27.0%) | NS |
| Middle (M) | 43(30.7%) | 49(40.2%) | |
| Lower (A) | 55(39.3%) | 40(32.8%) | |
| Borrmann | | | |
| Type 1 | 0(0%) | 1( 0.8%) | P<0.05 |
| Type 2 | 34(24.3%) | 24(19.7%) | |
| Type 3 | 57(40.7%) | 50(41.0%) | |
| Type 4 | 23(16.4%) | 35(28.7%) | |
| Type 5 | 26(18.6%) | 12(9.8%) | |
| Histology | | | |
| Differentiated | 52(37.1%) | 25(20.5%) | P<0.01 |
| Undifferentiated | 88(62.9%) | 97(79.5%) | |
| Prognostic serosal invasion (ps)[b] | | | |
| Negative | 44(31.4%) | 20(16.4%) | P<0.01 |
| Positive | 96(68.6%) | 102(83.6%) | |
| Histological lymph node metastasis | | | |
| Negative | 40(28.6%) | 21(17.2%) | P<0.05 |
| Positive | 100(71.4%) | 101(82.8%) | |
| Peritoneal dissemination | | | |
| Negative | 118(84.3%) | 94(77.0%) | NS |
| Positive | 22(15.7%) | 28(23.0%) | |
| Liver metastasis | | | |
| Negative | 133(95.0%) | 119(97.5%) | NS |
| Positive | 7(5.0%) | 3(2.5%) | |
| Operative procedure | | | |
| Partial | 80(57.1%) | 58(47.5%) | NS |
| Total | 60(42.9%) | 64(52.5%) | |
| Curability | | | |
| Curative | 92(65.7%) | 60(49.2%) | P<0.01 |
| Non-curative | 48(34.3%) | 62(50.8%) | |

[a]Mean ± standard deviation. [b]The definition of ps is given in Table I. NS, no significant difference.

**Table IV** Factors involved in non-curative resection in men and women under 50 years of age

| Variable | Men (n = 48) | Women (n = 62) |
|---|---|---|
| Serosal invasion | 1(2.1%) | 4(6.5%) |
| Lymph node metastasis | 10(20.8%) | 10(16.1%) |
| ow (+) | 5(10.4%) | 11(17.7%) |
| aw (+) | 4(8.3%) | 6(9.7%) |
| ow (+) and aw (+) | 2(4.2%) | 1(1.6%) |
| Peritoneal dissemination | 19(39.6%) | 27(43.6%) |
| Liver metastasis | 4(8.3%) | 2(3.2%) |
| Peritoneal dissemination and liver metastasis | 3(6.3%) | 1(1.6%0 |

ow (+) = cancer infiltration at the oral margin; aw (+) = cancer infiltration at the anal margin.

**Table V** Cox regression analysis of data on patients under 50 years of age

| *Covariate* | *P value* | *Relative risk* |
|---|---|---|
| Liver metastasis | <0.01 | 4.53 |
| Curability | <0.01 | 2.11 |
| Peritoneal dissemination | <0.01 | 1.78 |
| Operative procedure | <0.01 | 1.46 |
| Lymph node metastasis | <0.01 | 1.37 |
| Depth of invasion | <0.01 | 1.30 |
| Tumour maximal diameter | <0.01 | 1.05 |

**Figure 1** Survival curves for all patients of both sexes. Numbers of patients eligible for analysis at each point are shown.

tion could be fortuitous (Matley *et al.*, 1988). The presence of oestrogen receptors and intracytoplasmic oestradiol in a proportion of patients of all ages fails to explain the preponderance of women among young patients (Nishi *et al.*, 1987).

Armstrong and Dent (1986) reported that the survival rate of women with gastric cancer exceeds that of men, and Stemmermann and Brown (1974) reported that women with a diffuse gastric cancer had longer survival rates than did men. On the other hand, a diffuse type of gastric cancer which is relatively more frequent in women and young patients, results in a shorter survival time than seen with intestinal type or other types of histology (Laurén, 1965; Tso *et al.*, 1987). In our patients under age 50 years, the clinicopathological factors differed between the sexes, including factors of tumour size, Borrmann type, histology, depth of invasion, lymph node metastasis and operative curability. In particular, advanced cases were dominant in women and most often a non-curative resection was done. Therefore, the statistical lower survival rate for the women under age 50 years is expected to be influenced by operative curability, lymph node metastasis, depth of invasion and tumour size, all significant prognostic factors (Table IV). Clinical diagnosis may be made late and the young age of the women is the major deterrent to early diagnosis (Bloss *et al.*, 1980).

**Figure 2** Survival curves for men and women under 50 years of age. Numbers of patients eligible for analysis at each point are shown.

As tumour cells left behind at surgery may proliferate rapidly in non-curatively resected cases (Schabel, 1975; Gunduz *et al.*, 1979), the potential to control the remaining tumour foci is significantly reduced by postponing chemotherapy to the later postoperative period (Tubiana & Malaise, 1976; Douglass, 1985). We found that the undifferentiated gastric cancer tissue is more sensitive to anti-tumour drugs than is differentiated cancer tissue, *in vitro* (Maehara *et al.*, 1987) and the undifferentiated type is dominant in women under age 50 years. Therefore, postoperative chemotherapy, for example with the combination of mitomycin C, fluorinated pyrimidine and the immunomodulator PSK, should be initiated in a few days and continued for 1 year in the postoperative period (Maehara *et al.*, 1990*a*,*b*).

We thank M. Ohara for comments.

**References**

ARMSTRONG, C.P. & DENT, D.M. (1986). Factors influencing prognosis in carcinoma of the stomach. *Surg. Gynecol. Obstet.*, **162**, 343.

BABA, H., KORENAGA, D., OKAMURA, T., SAITO, A. & SUGIMACHI, K. (1989). Prognostic factors in gastric cancer with serosal invasion. Univariate and multivariate analyses. *Arch. Surg.*, **124**, 1061.

BLOSS, R.S., MILLER, T.A. & COPELAND, III, E.M. (1980). Carcinoma of the stomach in the young adult. *Surg. Gynecol. Obstet.*, **150**, 883.

BOKU, T., NAKANE, Y., OKUSA, T. & 5 others (1989). Strategy for lymphadenectomy of gastric cancer. *Surgery*, **105**, 585.

COX, D.R. (1972). Regression models and life tables. *J. Roy. Stat. Soc. Ser. B*, **34**, 187.

DIXON, W.J. (1985). *BMDP Statistical Software*. University of California Press: Berkeley.

DOUGLASS, H.O. Jr (1985). Gastric cancer: Overview of current therapies. *Semin. Oncol.*, **12**, 57.

GUNDUZ, N., FISHER, B. & SAFFER, E.A. (1979). Effect of surgical removal on the growth and kinetics of residual tumour. *Cancer Res.*, **39**, 3861.

JAPANESE RESEARCH SOCIETY FOR GASTRIC CANCER (1981). The General Rules for the Gastric Cancer Study in Surgery and Pathology. Part I. Clinical Classification. *Jpn. J. Surg.*, **11**, 127. Part II. Histological classification of gastric cancer. *Jpn. J. Surg.*, **11**, 140.

KODAMA, Y., SUGIMACHI, K., SOEJIMA, K., MATSUSAKA, T. & INOKUCHI, K. (1981). Evaluation of extensive lymph node dissection for carcinoma of the stomach. *World J. Surg.*, **5**, 241.

KORENAGA, D., TSUJITANI, S., HARAGUCHI, M. & 5 others (1988). Long-term survival in Japanese patients with far advanced carcinoma of the stomach. *World J. Surg.*, **12**, 236.

KORENAGA, D., HARAGUCHI, M., OKAMURA, T., BABA, H. & SUGIMACHI, K. (1989). DNA ploidy and tumor invasion in human gastric cancer. Histopathological differentiation. *Arch. Surg.*, **124**, 314.

LAURÉN, P. (1965). The two histological main types of gastric carcinoma: Diffuse and so-called intestinal-type carcinoma. *Acta Pathol. Microbiol. Scand.*, **64**, 31.

MAEHARA, Y., ANAI, H., KUSUMOTO, H. & SUGIMACHI, K. (1987). Poorly differentiated human gastric carcinoma is more sensitive to antitumor drugs than is well differentiated carcinoma. *Eur. J. Surg. Oncol.*, **13**, 203.

MAEHARA, Y., MORIGUCHI, S., SAKAGUCHI, Y. & 4 others (1990*a*). Adjuvant chemotherapy enhances long-term survival of patients with advanced gastric cancer following curative resection. *J. Surg. Oncol.*, **45**, 169.

MAEHARA, Y., WATANABE, A., KAKEJI, Y., BABA, H., KOHNOE, S. & SUGIMACHI, K. (1990*b*). Postgastrectomy prescription of mitomycin C and UFT for patients with stage IV gastric carcinoma. *Am. J. Surg.*, **160**, 242.

MAEHARA, Y., ORITA, H., MORIGUCHI, S. & 4 others (1991*a*). Lower survival rate for patients under 30 years of age and surgically treated for gastric carcinoma. *Br. J. Cancer*, **63**, 1015.

MAEHARA, Y., MORIGUCHI, S., YOSHIDA, M., TAKAHASHI, I., KORENAGA, D. & SUGIMACHI, K. (1991*b*). Splenectomy does not correlate with length of survival in patients undergoing curative total gastrectomy for gastric carcinoma. -Univariate and multivariate analyses. *Cancer*, **67**, 3006.

MAEHARA, Y., MORIGUCHI, S., KAKEJI, Y. & 4 others (1991*c*). Prognostic factors in adenocarcinoma in the upper one-third of the stomach. *Surg. Gynecol. Obstet.*, **173**, 223.

MATLEY, P.J., DENT, D.M., MADDEN, M.V. & PRICE, S.K. (1988). Gastric carcinoma in young adults. *Ann. Surg.*, **208**, 593.

MITSUDOMI, T., MATSUSAKA, T., WAKASUGI, K. & 5 others (1989*a*). A clinicopathological study of gastric cancer with special reference to age of the patients: An analysis of 1,630 cases. *World J. Surg.*, **13**, 225.

MITSUDOMI, T., TATEISHI, M., OKA, T., YANO, T., ISHIDA, T. & SUGIMACHI, K. (1989*b*). Longer survival after resection of non-small cell lung cancer in Japanese women. *Ann. Thorac. Surg.*, **48**, 639.

NISHI, K., TOKUNAGA, A., SHIMIZU, Y. & 6 others (1987). Immunohistochemical study of intracellular estradiol in human gastric cancer. *Cancer*, **59**, 1328.

SCHABEL, F.M. (1975). Concepts for systemic treatment of micrometastases. *Cancer*, **35**, 15.

SHIU, M.H., PERROTTI, M. & BRENNAN, M.F. (1989). Adenocarcinoma of the stomach: A multivariate analysis of clinical, pathological and treatment factors. *Hepato-gastroenterol.*, **36**, 7.

STEMMERMANN, G.N. & BROWN, C. (1974). A survival study of intestinal and diffuse types of gastric carcinoma. *Cancer*, **33**, 1190.

SUGANO, H., NAKAMURA, K., KATO, Y. (1982). Pathological studies of human gastric cancer. *Acta Pathol. Jpn.*, **32** (Suppl. 2), 329.

SUGIMACHI, K., MATSUOKA, H., MATSUFUJI, H., MAEKAWA, S., KAI, H., & OKUDAIRA, Y. (1987). Survival rates of women with carcinoma of the esophagus exceed those of men. *Surg. Gynecol. Obstet.*, **164**, 541.

THE GASTROINTESTINAL TUMOR STUDY GROUP (1982). Controlled trial of adjuvant chemotherapy following curative resection for gastric cancer. *Cancer*, **49**, 1116.

TSO, P.L., BRINGAZE, W.L., DAUTERIVE, A.H., CORREA, P. & COHN, I. Jr (1987). Gastric carcinoma in the young. *Cancer*, **59**, 1362.

TUBIANA, M. & MALAISE, E.P. (1976). Growth rate and cell kinetics in human tumors: Some prognostic and therapeutic implications. In *Scientific Foundations of Oncology*, Symington, T. & Carter, R.L. (eds). William Heinemann Medical Books: Chicago.